# Intelligence

**Published:** 1831-05

**Authors:** 


### INTELLIGENCE.

### ROYAL COLLEGE OF SURGEONS IN LONDON.

The printed Catalogue of the Library, comprising nearly 20,000 volumes, is ready for delivery to the members and articled students of the College, at the cost price, seven shillings. A Supplement will be printed annually, and the names of all donors of books will be duly recorded.

The library is open every day, except Sundays, from ten until four o'clock, from the 1st of October to the 1st of April, and from ten until five, during the remaining portion of the year; except on Saturdays, when it will be closed at one o'clock.

The Board of Curators being authorised by the College to publish, from time to time, whatever communications may be judged proper for publication, under the title of "Transactions of the Royal College of Surgeons in London," the members of the College, and other persons, are invited to make such communications to the Board, as may conduce to the improvement of anatomical or surgical science; and the manuscript of any communication which may be considered more proper for reference in the library, than for publication in the Transactions, will be preserved for that purpose.

EDMUND BELFOUR, Sec.

30*th March*, 1831.

*To the Editor of the London Medical and Physical Journal.*

*Apothecaries' Hall; April* 4*th*, 1831.

Sir: Numerous applications continuing to be made by gentlemen in almost every department of medical science, desiring to be recognised as lecturers by the Court of Examiners of the Society of Apothecaries, the Court feel anxious, in order to save the time now necessarily expended in correspondence with each successive applicant, that the rules which the Court have laid down for their own guidance in the recognition of the various courses of lectures required by them should become generally known to the profession: they have, therefore, desired me to transmit these rules to your Journal, with a request that you will be kind enough to give them an early insertion.

I have the honour to be, sir, your obedient servant,

JOHN WATSON,
Secretary to the Court of Examiners.

REGULATIONS *to be observed in the* RECOGNITION *of* LECTURERS, *extracted from the Minutes of the Court of Examiners, dated November* 18, 1830.

Resolved: That any person, being a member of the Court of Examiners shall not be recognised as a lecturer on any branch of medical science.

That the Court will not recognise any new teacher who may give lectures on more than *two* branches of medical science; nor will they sanction a teacher already recognised in giving lectures on any *new* branch of the science, if already he gives lectures on *two*.

That the Court will not recognise a teacher until he has given a public course of lectures on the subject he proposes to teach; but if, after such preliminary course of lectures, the teacher should be recognised, the student's certificate of attendance on that course will be received.

That the court will not recognise a teacher until he has produced very satisfactory testimonials of his attainments in the science he purposes to teach, and also of his ability as a teacher of it, from persons of acknowledged talents and distinguished acquirements in the particular branch of science in question.

That satisfactory assurance shall also be given that the teacher is in possession of the means requisite for the full illustration of his lectures; viz. that he has, if lecturing

On CHEMISTRY, a laboratory and complete apparatus;

On MATERIA MEDICA, a museum sufficiently extensive;

On ANATOMY and PHYSIOLOGY, a museum sufficiently well furnished with preparations, and the means of procuring recent subjects for demonstration;

On BOTANY, a Hortus Siccus, plates or drawings, and the means of procuring fresh specimens;

On MIDWIFERY, a museum, and such an appointment in a public midwifery institution as may enable him to give his pupils practical instructions.

That the lecturer on the PRINCIPLES and PRACTICE of MEDICINE must be, if he lectures in London or within seven miles thereof, a fellow, candidate, or licentiate of the Royal College of Physicians of London; and if he lectures beyond seven miles from London, and should not be thus qualified, he must be a graduated doctor of medicine of a British university of four years' standing, (unless, previously to his graduation, he had been for four years a licentiate of this Court.)

That the lecturer on MATERIA MEDICA and THERAPEUTICS must be a fellow, candidate, or licentiate of the Royal College of Physicians of London; a graduated doctor of medicine of a British university of four years' standing, (unless, previously to his graduation, he had been, for the same length of time, a licentiate of this Court;) or he must be a licentiate of this Court, of four years' standing.

That the lecturer on ANATOMY and PHYSIOLOGY must either be recognised by the Royal College of Surgeons of London, or must be a member of that College, of four years' standing.

That the demonstrator of ANATOMY must either be recognised by the Royal College of Surgeons of London, or must be a member of that College.

---

LIABILITY *of* MEDICAL MEN *to* SERVE *on* JURIES.

Our correspondent, "A Physician," is right, and the magistrate referred to is wrong. The former asserts that medical practitioners are *not* liable to serve on juries: by the latter it has been maintained that they have no such exemption in law. It is expressly stated by Mr. WILLCOCK, (Laws of the Medical Profession, page 137,) that neither physicians, surgeons, being members of the London, Edinburgh, or Dublin Colleges, nor legally constituted apothecaries, can be called upon to serve as jurymen. Mr. W. refers to the various statutes upon the point. It appears, therefore, that the magistrate permitted as a favor what the practitioner might have demanded as his right.

---

KING'S COLLEGE, LONDON.

Among other appointments which have recently been made in this Institution, we perceive that Mr. G. T. Burnett has been elected Professor of Botany. The appointment of this gentleman affords an additional proof of the judgment with which the professors of the various branches of science are selected.

---

### LITERARY NOTICE.

Mr. Wallace, surgeon to the Charitable Infirmary, and to the Infirmary for Diseases of the Skin, Dublin, will shortly publish the History and Treatment of Venereal Diseases of the Skin, including the Primary Symptoms and Eruptions; illustrated by delineations as large as life, and coloured after nature.

---

### MONTHLY LIST OF MEDICAL BOOKS.

### METEOROLOGICAL JOURNAL,

**By Messrs. Harris and Co. Mathematical Instrument Makers, 50, High Holborn.**

| | Moon. | Rain gauge. | Thermom. | | | Barometer. | | De Luc's Hygrometer. | | Winds. | | Atmospheric Variation. | | |
|---|---|---|---|---|---|---|---|---|---|---|---|---|---|---|
| Mar | | | *9 a.m.* | *max.* | *min.* | 9 *a.m.* | 10 *p.m.* | 9 *a.m.* | 10*p.m.* | 9 *a.m.* | 10*p.m.* | 9 *a. m.* | 2 *p.m.* | 10 *p.m.* |
| 20 | ☽ | .27 | 50 | 52 | 45 | 29.96 | 29.90 | 56 | 59 | W | NW | Fine | Fine | Fine |
| 21 | | | 51 | 53 | 43 | .93 | .98 | 59 | 60 | NNE | ESE | — | — | — |
| 22 | | | 45 | 46 | 39 | 30.11 | 30.13 | 61 | 58 | ESE | NE | Cloudy | — | — |
| 23 | | | 44 | 44 | 32 | .16 | .12 | 55 | 57 | E | E | Fine | — | Cloudy |
| 24 | | | 35 | 44 | 34 | 29.79 | 29.62 | 59 | 59 | NE | ENE | Sleet | Cloudy | — |
| 25 | | | 42 | 45 | 44 | .51 | .41 | 58 | 64 | SE | E | Fine | Fine | — |
| 26 | | | 48 | 49 | 41 | .33 | .47 | 64 | 68 | ESE | S | Rain | — | Fine |
| 27 | | .16 | 46 | 57 | 44 | .72 | .81 | 67 | 63 | | S | Fine | — | — |
| 28 | ○ | | 50 | 57 | 40 | .90 | .94 | 62 | 62 | ESE | ESE | — | — | — |
| 29 | | | 43 | 46 | 39 | 30.02 | 30.07 | 60 | 58 | ENE | NE | Cloudy | — | — |
| 30 | | | 44 | 50 | 35 | .13 | .15 | 55 | 55 | ENE | NE | Cloudy | — | Cloudy |
| 31 | | | 42 | 46 | 38 | .22 | .30 | 54 | 51 | ENE | ENE | Fine | — | — |
| *April* | | | | | | | | | | | | | | |
| 1 | | | 44 | 47 | 37 | 30.18 | 29.96 | 53 | 54 | ENE | NE | — | — | Sho'ry |
| 2 | | | 42 | 43 | 38 | 29.90 | .87 | 57 | 62 | E | NE | Cloudy | — | Cloudy |
| 3 | | | 44 | 50 | 39 | .74 | .60 | 62 | 62 | NE | NE | Fine | — | Fine |
| 4 | | | 45 | 48 | 37 | .52 | .42 | 62 | 62 | NE | S | Cloudy | Cloudy | Cloudy |
| 5 | ☾ | | 49 | 52 | 42 | .33 | .32 | 62 | 60 | W | W | — | Fine | Fine |
| 6 | | | 53 | 57 | 43 | .33 | .31 | 60 | 59 | W | SW | Fine | — | — |
| 7 | | | 54 | 60 | 49 | .16 | .11 | 56 | 58 | SE | S | — | Cloudy | Cloudy |
| 8 | | | 50 | 57 | 55 | .11 | .23 | 60 | 64 | svar. | SW | Sho'ry | Rain | — |
| 9 | | .55 | 57 | 62 | 48 | .34 | .36 | 62 | 60 | SW | S | Cloudy | Fine | — |
| 10 | | | 53 | 59 | 43 | .49 | .71 | 61 | 61 | SW | SW | — | — | Fine |
| 11 | | | 49 | 57 | 50 | .80 | .84 | 60 | 60 | NNW | SE | Fine | Cloudy | — |
| 12 | ● | | 58 | 62 | 49 | .72 | .65 | 60 | 63 | ENE | ESE | Fine | Fine | Rain |
| 13 | | | 62 | 64 | 47 | .65 | .66 | 63 | 61 | ESE | NNE | Cloudy | — | Fine |
| 14 | | | 51 | 62 | 45 | .80 | .79 | 61 | 58 | NNE | SW | — | — | — |
| 15 | | | 52 | 57 | 52 | .84 | .85 | 58 | 55 | NW | NW | Fine | — | — |
| 16 | | | 56 | 59 | 44 | .87 | .85 | 58 | 57 | NNE | NE | Cloudy | — | — |
| 17 | | | 52 | 55 | 39 | .85 | .85 | 57 | 56 | | NE | Sho'ry | Cloudy | — |
| 18 | | .20 | 50 | ç6 | 41 | .87 | .86 | 56 | 56 | NE | NE | Fine | Fine | — |
| 19 | ☽ | | 46 | 51 | 40 | .79 | .74 | 57 | 57 | NE | NE | Cloudy | — | — |

**The quantity of Rain fallen, in March, was 1 inch and 61,100ths.**

